# A systematic review of the measurement properties of self-report screening tools to detect risk or exposure to child sexual abuse for children under 12

**DOI:** 10.1016/j.heliyon.2023.e21027

**Published:** 2023-10-21

**Authors:** Karla Danielle Xavier do Bomfim, Umbelina do Rego Leite, Paulo Sávio Angeiras de Goes

**Affiliations:** a Post-graduate Program on Child and Adolescent Health, Universidade Federal de Pernambuco (UFPE), Recife, Pernambuco, Brazil; b Psychology Department, Universidade Federal de Pernambuco (UFPE), Recife, Pernambuco, Brazil

**Keywords:** Systematic review, Validation as topic, Child sexual abuse, Domestic violence, Sexual violence, Risk, Secondary prevention

## Abstract

Child sexual abuse (CSA) is a substantial public health issue that is challenging to measure epidemiologically due to the “pact of silence” among those involved. Validated tools could contribute to early recognition or risk detection for CSA. We aimed to systematically assess self-report tools’ measurement properties and methodological quality that detect risk or exposure to CSA for children under twelve. The search strategy, selection criteria, data extraction, data analysis, and synthesis followed the COSMIN methodology for systematic reviews (2018). PROSPERO 2021 registration CRD42021278465. MEDLINE, COCHRANE, EMBASE, CINAHL, SCOPUS, and ERIC were searched until August 2021, with an updated search on September 23, 2021, and unlimited by language. The inclusion criteria were: to assess risk or exposure to CSA under twelve years old by objective items and self-report tools; sexual violence risk or exposure in the domestic context; the application context should include health facilities (such as hospital emergency rooms, outpatient clinics, pediatric wards, psychology centers, social services), education (such as schools) and community; no language or date restriction. The exclusion criteria were: non-self-report tools studies, comprehensive articles, comments, editorials, expert opinions, and studies of projective techniques. The COSMIN risk of bias checklist was used to evaluate the methodological quality of the included studies. Feasibility aspects were assessed. This study had no funding source; 29 studies describing eight tools met eligibility criteria. No single instrument reported all nine measurement properties outlined by the COSMIN methodology. The strength of the evidence was moderate to high for six out of eight instruments. ICAST-C and JVQ were the tools that obtained the highest number of rated measurement properties and strength of evidence.

## 1 Introduction

Child sexual abuse (CSA) is a major public health problem difficult to measure epidemiologically due to the “pact of silence” among those involved. It is more common in children under 12 and the domestic setting is the most frequent context. The overall CSA estimated prevalence rate is 127/1000 children. The self-reported prevalence of CSA victimization is likely more than 30 times higher than official reports [[1], [2], [3], [4], [5], [6]].

CSA is an adverse childhood event (ACE) that involves severe consequences to intergenerational biopsychosocial health, and it is associated with the co-occurrence of other types of violence throughout life [[7], [8], [9], [10]]. The disclosed cases taken to the pediatric emergency rooms or outpatients clinics are probably only the tip of the iceberg [11,12]. In contexts where there are high levels of community violence, local health and education professionals could be reluctant to report suspicious cases [13,14].

Using validated tools could contribute to early detection or risk for domestic CSA. The domestic context is more prevalent in reported cases of CSA, and it involves abuse cycles across generations [4,6,7]. Detecting risks before exposure is a secondary prevention challenge that potentially may prevent deleterious biopsychosocial effects.

There is significant literature published on screening tools for adults and adolescents regarding situations of CSA that occurred in early life [[15], [16], [17], [18], [19]]. Screening risks or exposure still in childhood could mitigate the time dependent effects of CSA in health and its epigenetic influence on germ cells [20]. Co-occurrence between CSA and other violence in childhood and throughout life can be as high as 50 % [9]. As CSA has a considerable co-occurrence with other forms of violence, it is possible to infer that the earlier the detection and preventive action, the healthier the individual will be throughout life [21,22]. A US$ 1.05 × 10^14^ annual economy with a 10 % ACE prevalence reduction is estimated [23]. These are pertinent justifications for focusing this study on children under 12, regardless of the prevalence estimates in this age group. In addition, it is important to understand how much adaptation to child developmental perspective has been considered for instruments’ development.

Saini et al. (2019) [15] was the first systematic review that summarized measurement properties and methodological quality of published child abuse tools using COnsensus‐based Standards for the selection of health Measurement INstruments’ (COSMIN) checklist (Mokkink et al., 2010) [24]. It focused on one or more of the five main subtypes of child abuse by caregivers, including sexual abuse, physical abuse, emotional abuse and neglect, providing information on the developmental timing of child abuse. Most of the 52 tools reviewed were adult self-report and retrospectively measured child abuse before the age of 18. Only eight instruments developed for 18 years of age and older showed strong to moderate levels of evidence, but not for all nine COSMIN criteria [15].

This current systematic review focuses on domestic CSA in children under 12 because it is one of the most challenging forms of violence for professionals to perceive, and it has substantial repercussions on biopsychosocial and intergenerational health from a developmental perspective. Domestic CSA exposure is a sexual abuse experience in domestic violence context. It includes the homes in which children usually go, and it may also comprehend extra-familial CSA. The domestic violence term in a broader definition includes all documented forms: sexual assault/abuse, emotional/psychological, physical violence, controlling/threatening behavior, and coercion between intimate partners and towards children (both within and outside the household by a parent/primary caregiver, biological or non-biological) [29].

**Fig. 1:**
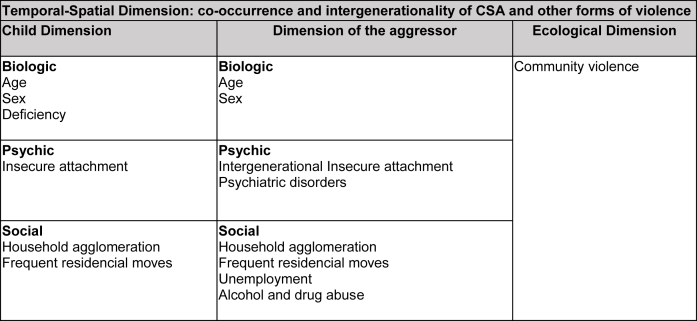
Our inferred risk model for domestic CSA.

Risk for CSA is not a delimited construct in scientific literature. But there are reported risk factors: violence intergenerationality [7,30], insecure attachment to parents [22,29], physical and psychological domestic violence [9,12], caregivers' alcohol abuse, and community violence [4,6]. Studies are needed for an approximation to this complex pre-exposure context. It is recommended that researchers account for violence co-occurrence in explanatory models [9]. Fig. 1 shows our inferred risk model for domestic CSA, which includes dimensions of the child and the aggressor, from a spatial-temporal (co-occurrence and intergenerationality) and biopsychosocial perspective.

Thus, this review aims to provide an overview of the quality of all available self-report tools for children under 12 years of age that can screen for risk and/or exposure to domestic CSA**.** It may help to identify under-12 y children's self-reported instruments with better feasibility and quality of measurement properties.

To review screening tools for CSA in under-12 y children, some considerations were necessary for the choice of tools: (1) self-report tools were chosen (self-administered or interview-based), as literature has found different correlations observed between the reports of caregivers and children, in which the children's reports were often more representative of maltreatment [[25], [26], [27], [28]]; (2) the tools should contain items that screen for risk or exposure situations for CSA. Risk situations should encompass one or more of some significant risk factors for CSA: physical violence, psychological violence (including witnessing domestic violence), and neglect [29,30]. Exposure situations would include victimization, however not including screening tools for psychosocial harms associated with CSA or other types of violence (e.g., post-abuse adjustment, depression, post-traumatic distress), as this systematic review intended to contribute to a secondary risk prevention or early detection approach. This would contribute to identifying families who should be monitored for risk situations or investigated for exposure to CSA.

### 1.1 Search strategy and selection criteria

This review employed the updated Consensus-based Standards for the Selection of Health Measurement Instruments (COSMIN) methodology for systematic reviews of PROMs [31,32]. This work participated in the PRISMA-COSMIN guideline pilot testing when performing adjustments recommended by peer review [63].

Studies of the measurement properties and those assessing the methodological quality of the tools were included. Only self-report (self-administered or by interview) tools that measure risk or exposure to CSA under-12 y children were included. Tools for screening trauma related disorder or symptoms (e.g., post-traumatic stress disorder, dissociative disorder, depression) were not included, because this systematic review intends a more preventive or early detection approach.

The inclusion criteria were.

(1) The screening tool for the literature should include the following items:
  (a) to assess risk or exposure to CSA on children under twelve years old by objective items and self-report tools; (b) the sexual violence risk or exposure context is domestic; (c) the screening tool is suitable for children under the age of 12; (d) the application context should include health places (such as hospital emergency, out-patient clinics or pediatric wards, psychology, social worker), education (such schools) and community.
(2) No language restriction.
(3) No date restriction.

The exclusion criteria were: non-self-report tools studies, comprehensive articles, comments, editorials, expert opinions, studies of projective techniques.

The risk context screening tools should address in their categories psychological, physical or neglect violence, which are considered as risk factors for CSA. Exposure to maternal intimate partner violence falls under the category of psychological violence.

A systematic search was performed in six electronic databases: MEDLINE; COCHRANE; EMBASE; CINAHL; Scopus and ERIC. We reviewed all existing full-text articles published until August 2021 with an updated search on September 23, 2021 and unlimited by language. Reference lists of included studies and related articles were checked. This systematic review used additional sources for search blocks found on the website https://blocks.bmi-online.nl/, as recommended in COSMIN [31]. The following terms were used: child* OR pediatric* OR infant* OR kid* OR boy OR boys OR girl* OR paediatric* OR pediatry OR infanc* OR infant*; “Self Report”; “early life”; (Self AND Report*) AND (tool* OR instrument* OR apparatus OR device* OR equipment*); “Sex Offenses” OR “Child Abuse, Sexual”; ((Violence* OR injur* OR abuse* OR offense*) AND (Sexual OR sex)); “Family Relations” OR Family; home OR house* OR famil* OR dependants OR dependents OR domestic OR filiation OR Relatives OR Stepfamilies OR Stepfamily OR intrafamilial OR (intra AND famil*) OR interparent* OR intergenerational; “Risk-Taking” OR “Risk Factors” OR “Exposure to Violence”; risk OR OR danger OR hazard OR imminence OR menace OR peril OR pitfall OR threat OR trouble OR exposure; “validation study” OR methods OR “comparative study” OR psychometrics OR “outcome assessment” OR “observer variation” OR “health status indicators” OR “reproducibility of results” OR “discriminant validation”; (instrumentation OR psychometr* OR clinimetr* OR clinometr* OR “outcome assessment” OR “outcome measures” OR “outcome measure” OR “observer variation” OR reproducib* OR reliab* OR unreliab* OR valid* OR coefficient OR homogeneity OR homogeneous OR “internal consistency” OR (cronbach* AND (alpha OR alphas)) OR (item AND (correlation* OR selection* OR reduction*)) OR agreement OR precision OR imprecision OR “precise values” OR “test– retest” OR (test AND retest) OR (reliab* AND (test OR retest)) OR stability OR interrater OR “inter-rater” OR intrarater OR “intra-rater” OR intertester OR “inter-tester” OR intratester OR “intra-tester” OR interobserver OR “inter-observer” OR intraobserver OR intertechnician OR “inter-technician” OR intratechnician OR “intra-technician” OR interexaminer OR “inter-examiner” OR intraexaminer OR “intra-examiner” OR interassay OR “inter-assay” OR intraassay OR “intra-assay” OR interindividual OR “inter-individual” OR intraindividual OR “intra-individual” OR interparticipant OR “inter-participant” OR intraparticipant OR “intra-participant” OR kappa OR “kappa's" OR kappas OR repeatab* OR ((replicab* OR repeated) AND (measure OR measures OR findings OR result OR results OR test OR tests)) OR generaliza* OR generalisa* OR concordance OR (intraclass AND correlation*) OR discriminative OR “known group” OR “factor Analysis” OR “factor analyses” OR dimension* OR subscale* OR (multitrait AND scaling AND (Analysis OR analyses)) OR “item discriminant” OR “interscale correlation” OR “interscale correlations” OR error OR errors OR “individual variability” OR (variability AND (Analysis OR values)) OR (uncertainty AND (measurement OR measuring)) OR “standard error of measurement” OR sensitiv* OR responsive* OR ((minimal OR minimally OR clinical OR clinically) AND (important OR significant OR detectable) AND (change OR difference)) OR (small* AND (real OR detectable) AND (change OR difference)) OR “meaningful change” OR “ceiling effect” OR “floor effect” OR “Item response model” OR IRT OR Rasch OR “differential item functioning” OR DIF OR “computer adaptive testing” OR “item bank” OR “cross-cultural equivalence").

The gray literature sources were also assessed. The search only found articles in English. Some study authors were contacted to provide additional information. Unpublished studies were included.

The data extraction also followed COSMIN methodology for systematic reviews of Patient Reported Outcome Measures [31]. The recommended Covidence® tool was used and automatically excluded the duplicate data. Titles and abstracts identified by the search strategies were screened by two independent reviewers (KDXB and URL). Full-text copies of the relevant articles were assessed. Disagreements were resolved by the judgment of the third reviewer (PSAG). The study protocol is registered in PROSPERO 2021 CRD42021278465. Available in: https://www.crd.york.ac.uk/prospero/display_record.php?ID=CRD42021278465.

The authors declare a conflict of interest because they are developing a risk detection tool for domestic sexual violence for 9-11-year-old schoolchildren.

### 1.2 Data extraction

COSMIN methodology for systematic reviews of Patient‐Reported Outcome Measures [30] was used to assess the quality of studies on nine measurement properties (content validity, internal consistency, reliability, structural validity, hypothesis testing, cross-cultural validity, criterion validity, measurement error and responsiveness) and general design requirements for tool development ─ clear origin and description of the construct, target population representation, qualitative methods used, pilot test, comprehensibility, comprehensiveness. The methodological quality of the included studies was assessed by two reviewers independently (KDXB and URL) and discrepancies were solved by the third reviewer (PSAG). Risk of bias could occur if the quality of the study was inadequate or doubtful, as assessed with the COSMIN Risk of Bias tool to assess the quality of studies on reliability and measurement error of outcome measurement tool [32], or if only one study of adequate quality was available.

Feasibility did not refer to a quality parameter, but it was important to be taken into consideration in the selection of the most appropriate tool for the target age of children. Feasibility aspects included: patient's comprehensibility, type and ease of administration, length of the tool, completion time, patient's required mental level/physical ability and other items (Table 1).

**Table 1:** Feasibility of selected self-report instruments for children under 12.

Instrument	Children's comprehensibility	Type of administration, length of the instrument and completion time	Availability in different settings and children's required mental and physical ability level	Cost of an instrument and regulatory agency's requirement for approval
**CTSPC** *The Parent-Child Conflict* *Tactics Scale* [[33], [34], [35]] It assesses both current violent and non-violent techniques, and provides information on four disciplinary categories: (1) non-violence, (2) psychological aggression, (3) corporal punishment, and (4) physical abuse. The CTSPC includes a supplemental scale on neglect and additional questions on corporal punishment and sexual abuse.	The original CTS was designed to be used with partners. Twenty-two studies were based on the administration of the CTS1 to children ranging from 6 to 17 years old. CTSPC Sinhala version was tested in 12-year-old children.	Interview **or** self-administered 64 questions for 16 items; 32 questions long if used by and towards just one of the parents. Approximately 10 Minutes for completion	For research and clinical settings. For children aged 6 to 17.	Introductory Kit cost €90 (handbook, 10 forms), €90 (25 forms) Permission to use it must be obtained from WPS Western Psychological Services (WPS) www.wpspublish.com
**JVQ** *Juvenile Victimization Questionnaire* [[36], [37], [38], [39]] It provides screening questions about 34 current offenses against youth which cover five general areas of concern: (1) Conventional Crime, (2) Child Maltreatment, (3) Peer and Sibling Victimization, (4) Sexual Victimization, and (5) Witnessing and Indirect Victimization.	Children's ability to comprehend survey items was assessed and refined through a series of cognitive interviews with two dozen children, aged 6–15. This tool was designed to have two formats. One, for ages 8 to 17 (child self report, interview setting), and the other for 2 to 8-year-olds (parent report). It could be self-administered for 12 and older.	Interview format (face-to- face or over the telefone). . 37-item 40-item (JVQ-R2, 2nd Revision) 20 min for completion.	For clinical, research, and community settings. Highly verbal 6- and 7-year-old children may be able to complete the child self-report.	Free online. Regulatory agency: Crimes Against Children Research Center, Durham, NH: http://www.unh.edu/ccrc/jvq/available_versions.html kelly.foster@unh.edu
**ICAST- C** *International Association* *for the Prevention of* *Child Abuse and Neglect* *Child Abuse Screening* *Tool* [[40], [41], [42], [43]] The ICAST-CH measures a child's current (past-year) and lifetime exposure to physical, emotional and sexual abuse, neglect, domestic violence and community violence. The ICAST-CI measures victimization at school or other institutional environments. ICAST-CH and ICAST-CI were consolidated in 2016 into one ICAST-C version. It was designed to study the prevalence of childhood victimization.	Children's ability to comprehend survey items was assessed and refined through a series of cognitive interviews with two dozen children, aged 6–15. This tool was designed to have two formats. One, for ages 8 to 17 (child self report, interview setting), and the other for 2–8 year olds (parent report). It could be self-administered for 12 and older.	Self-administered The original ICAST-C has 38 items. That number is often modified, depending on the country in which the tool is employed. 10–30 min for completion	For research and clinical settings Study investigators felt that children under 12 were not emotionally mature enough for the content of the survey (pilot tests included inadvertently children under 12 in the convenience sample).	Free online Regulatory agency: ISPCAN *International Association* *for the Prevention of* *Child Abuse and Neglect* resources@ispcan.org
**DDI-C (form C)** Dimensions of Discipline *child self-report, form C* [[44], [45], [46], [47]] It provides current information on 26 of the most frequently used discipline behaviors of parents, measuring the frequency of using: • Corporal punishment; • Deprivation of privileges; • Diversion; • Explain/teach; • Ignore misbehavior; • Penalty tasks and restorative behavior; • Psychological aggression; • Reward; • Monitoring; • Context: conflict with partner, confidence, perceived ineffectiveness of disciplinary measures, stress; • Sociodemographic risk scale.	It simplified the wording of some items, however depending on the age of children and the sample, further modifications could be necessary.	Interview or self-administered 77-item 10–20 min for completion.	For research and clinical settings Self-administered for children aged 10 to 12; authors suggested an interview for children 6 to 9, using a one-month reference period.	Free online Regulatory agency: Straus & Fauchier Family Research Laboratory, University of New Hampshire 126 Horton SSC, Durham, NH 03824 angele.fauchier@unh.edu. PHONE: 603-862-2594 FAX: 603-862-1122 http://pubpages.unh.edu/∼mas2
**TISH** *Things I've seen and* *heard* [[48], [49], [50]] It measures current types of violence both witnessed and directly experienced by children, and examines the extent to which these exposure patterns are related to characteristics of the children and their families.	It uses illustrations to facilitate comprehension. For each description of violence, there are five stacks of balls labeled sequentially from “never" to “many times”. Children were asked to circle a stack of balls indicating their frequency perception.	Interview 20-item 5–10 min for completion	For research and clinical settings It assessed children aged 6 to 12	Free from Developer Regulatory agency: John Richters, Child and Adolescent Disorders Research Branch, Division of Clinical Research, National Institute of Mental Health, 5600 Fishers Lane, Room 10–104, Rockville, MD 20857
**MNBS-CR** *Multidimensional* *Neglectful Behavior Scale- Child Report* [[51], [52], [53], [54]] It measures retrospective and current neglect in four dimensions for the basic needs of development in four 5-item subscales: (1) neglect of physical needs, such as food, clothing, shelter, medical care; (2) emotional needs such as affection, companionship, support; (3) supervisory needs such as setting limits, attending to misbehavior, knowing a child's whereabouts and friends; and (4) cognitive needs such as reading to the child, and explaining things.	MNBS-CR is a computer administered interviewing system with questions based on the child's age, with 2 versions: younger, from 6 to 9, and older, from 10 to 15 years old.	Self-administered or interview-guided, picture-based for younger children 66-item There are two separate forms for children between the ages of six and nine and 10–15 year-olds Up to 20 Minutes for completion	For research and clinical settings Children's versions are pictorial and do not require reading ability	Free online Regulatory agency: Family Research Laboratory, University of New Hampshire 126 Horton SSC, Durham, NH 03824
**C-SARS** Checklist of *Sexual Abuse* *and Related Stressors* [55,56] It assesses victims' reports of retrospective occurrence of 70 stressful events related to sexual abuse experiences. .	The interviewer could answer questions about the items on the interview to monitor negative emotional signs related to the interview.	Interview 350-item 90–120 min for completion	For research and clinical settings It is suitable for 11–18 years old children	Free from Developer Regulatory agency: Institute for Juvenile Research, University of Illinois at Chicago. Steve Spaccarelli, Department of Psychiatry, University of Illinois at Chicago, 9075 Wolcett Ave., Chicago, IL 60612
**CEDV** *Child Exposure to Domestic Violence Scale* [[57], [58], [59], [60], [61]] It assesses current types of adult domestic violence to which a child is exposed, how a child is exposed to each, how the child is involved in violent incidents as well as information on other forms of victimization, and risk and protective factors.	Researchers changed words and sentence structures to adapt to the reading level of the interviewed children; answers were simplified by framing questions.	Self-administered 46-item 20 min for completion	For research and clinical settings For children aged 9–16 years old .	Free online Regulatory agency: Minnesota Center Against Violence and Abuse School of Social Work mincava@umn.edu

**Fig. 2:**
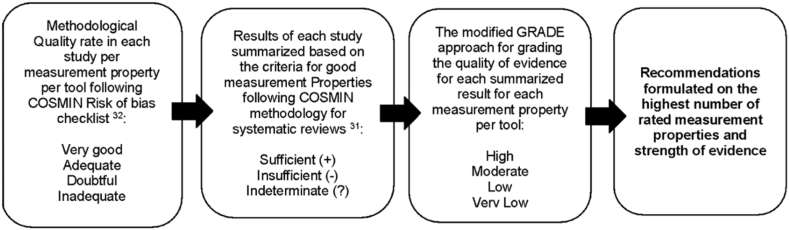
Steps to summarize methodological quality and strength of evidence of the assessed instruments.

Instruments development and content validity are not measurement properties. To rate content validity and grade the quality of evidence, it is necessary a subjective judgment of the tool development, the studies on content validity, the instrument itself, and the quality of total body of evidence. We followed COSMIN risk of bias checklist [32] to assess requirements and COSMIN methodology for assessing the content validity [65] to grade the quality of evidence on content validity. The methodological quality was rated in each study per measurement property per tool (i.e., very good, adequate, doubtful, inadequate) following the COSMIN risk of bias checklist [32]. The results of each study, and the accompanying ratings of the results were qualitatively summarized, based on the updated criteria for good measurement properties (sufficient (+)/insufficient (‐)/indeterminate (?) [31]. The modified GRADE approach for grading the quality of evidence (high, moderate, low, very low) was used to determine strength of evidence for each summarized result for each measurement property per tool [31] (Table 2). The criteria for quality rating of measurement properties is also described in Table 2. The quality of the evidence was not graded for indeterminate (?) overall rating studies. GRADE approach evaluations were performed by two reviewers independently. The third reviewer was consulted to reach a consensus. To formulate recommendations. We considered the results on the highest number of rated measurement properties and strength of evidence. These steps to summarize methodological quality and strength of evidence of the assessed instruments are in Fig. 2.

**Table 2:** Quality of the psychometric properties of each included study and evidence quality per self-report instrument for under-12 y children.

**Tool**	**Citation**	**Instrument Development**^a^ *Very good general design requirements: clear origin and description* of the *construct, target population representation, qualitative methods used, pilot test, comprehensibility, comprehensiveness* *Tool development is not a measurement property; it accounts when evaluating content validity*	**Content validity** *Very good content validity requirements: asking patients and professionals about the relevance,* *comprehensiveness and comprehensibility of the tool*	**Internal consistency** Sufficient overall rating (+): *Cronbach's alpha(s) ≥ 0.70 for each unidimensional scale or subscale AND at least low evidence for sufficient structural validity*	**Reliability** Sufficient overall rating (+): *ICC or weighted Kappa ≥ 0.70*	***Structural validity*** Sufficient overall rating (+): *CFA (confirmatory factor analysis: CFI (comparative fit index) or TLI (Tucker‐Lewis* *index or comparable measure* > *0.95) OR RMSEA (Root Mean Square Error of Approximation* <*0.06) OR SRMR (Standardized Root Mean Residuals) < 0.082′*	***Hypothesis testing*** Sufficient overall rating (+): *The result is in accordance with the hypothesis*	***Criterion validity*** Sufficient overall rating (+): *Correlation with gold standard ≥ 0.70 OR AUC (area under the curve) ≥ 0.70*	***Cross-cultural validity*** Sufficient overall rating (+): *No important differences found between group factors (such* *as age, gender, language) in multiple group factor analysis OR* *no important DIF (differential item functioning, for group factors) (McFadden's R2* < *0.02)*	**Measurement error** Sufficient overall rating (+): *SDC (smallest detectable change) or LoA (limits of agreement) < MIC (minimal important change)*	***Responsiveness*** Sufficient overall rating (+): *The result is in accordance with the hypothesis OR AUC (area under the curve) ≥ 0.70*

**CTSPC** *The Parent-Child Conflict* *Tactics Scale* [[33], [34], [35]] It assesses both current violent and non-violent techniques, and provides information on four disciplinary categories: (1) non-violence, (2) psychological aggression, (3) corporal punishment, and (4) physical abuse. The CTSPC includes a supplemental scale on neglect and additional questions on corporal punishment and sexual abuse	**Straus et al**, **1998** [33] **Straus et al**, **2006** [34]	**Adequate**^a^ Revision to develop CTSPC, an even more useful instrument than the CTS1; 22 studies were based on administration of the CTS1 to children, ages 6–17; the theoretical basis is the same for both tools; the wording of all items was reviewed to improve clarity and appropriateness; the interviews by telephone were pretested on a sample of 14 cases; 1000 interviews were completed in a probability sample of telephone households in the continental US. Some aspects were not clearly described (skilled group moderators/ interviewers, use of interview guide/interviews recorded and transcribed, qualitative methods for analyzing the data, number of researchers coding the data)		**Q** = **(−)** Overall alfa = .55			**Q** = **(** + **)** Positive correlations between scales with all hypothesis				
	**De Zoysa et al**, **2005** [35] 111 schoolchildren, age 12, enrolled to pilot-testing in Sri-Lanka Internal consistency and test-retest reliability assessed by a large group (entire class, about 40 children) and small group (about 20 children)		**Adequate**^a^ Based on two rounds of Delphi process with six experts the Sinhala version was first pre-tested with five schoolchildren aged 12 y. A second feedback of a 12-year-old children focus group was used to further revise the instrument. Five items were modified to make them culturally appropriate to be used in Sri Lanka; 111 12 y schoolchildren were enrolled to pilot-testing. Some aspects were not clearly described (use of interview guide, number of researchers coding the data)	**Q** = **(** + **)** Overall alfa: large group = .81; small group = .95	**Q** = **(−)** 14- day test-retest ICC: large group = .62; small group = .88						

***Evidence quality per measurement property***	**NA**	**NA**	**QE: high**	**QE: moderate**						

**JVQ** *Juvenile Victimization Questionnaire* [[36], [37], [38], [39]] It provides screening questions about 34 current offenses against youth that cover five general areas of concern: (1) Conventional Crime, (2) Child Maltreatment, (3) Peer and Sibling Victimization, (4) Sexual Victimization, and (5) Witnessing and Indirect Victimization	**Hamby et al**, **2004** [36]	**Adequate**^a^ Cognitive interviews with 24 schoolchildren aged 6 to 15. Some aspects were not clearly described (skilled group moderators/ interviewers, use of interview guide/interviews recorded and transcribed, qualitative methods for analyzing the data, number of researchers coding the data)									

	**Finkelhor et al**, **2005** [37] 2030 children ages 2–17, in contiguous United States.			**Q** = **(** + **)** Overall Cronbach's alpha = 0,8	**Q** = **(−)** 100 self-respondents Mean *k* = 0.63			**Q** = **(−)** Correlations with Trauma Symptoms Checklist for Children (TSCC) < 0,7 (N = 922)			

**Pereda et al, 2016** [38,39] 804 adolescents, ages 12–17, in seven secondary schools in northeastern Spain					**Q** = **(** + **)** RMSEA = .053 SRMR = .012					

***Evidence quality per measurement property***	**NA**		**QE: high**		**QE: high**		**QE: high**			

**ICAST- C** *International Association* *for the Prevention of* *Child Abuse and Neglect* *Child Abuse Screening* *Tool* [[40], [41], [42], [43]] The ICAST-CH measures a child's current (past-year) and lifetime exposure to physical, emotional and sexual abuse, neglect, domestic violence and community violence. The ICAST-CI measures victimization in school or other institutional environments. ICAST-CH and ICAST-CI were consolidated in 2016 into one ICAST-C version. It was designed to study the prevalence of childhood victimization.	**Zolotor et al**, **2009** [40] ICAST-CH and ICAST –CI	**Adequate**^a^ The study used a group of experts from 40 countries to design a survey by consensus to study the prevalence of childhood victimization. Two Delphi rounds were necessary. Authors asked each site to conduct at least one focus group with schoolchildren prior to field testing the instrument. There is a lack of information about comprehensibility and comprehensiveness.		**Q: (−)** Alfa ranged .72–.86, with the exception of the violence exposure scale (alpha = .69) Sample size (n = 571) ranged from 110 to 122 schoolchildren in four countries (Columbia. India, Russia, Iceland); ages from 11 to 18 years (34 children under 12 y)							

	**Chang et al, 2013** [41] ICAST–CH–C (chinese version of ICAST-CH)			**Q: (−)** 98 adolescents in grades 7–12 (mean age = 14.5; S.D. = 1.32) Overall alfa = 0,90 Cronbach's alphas for the five subscales ranged from .71 to .89 Item-subscale correlations: .53 to .82 (p < .01)		**Q: (−)** RMR = 0,27 RMSEA = 0,63 GFI = 0,825 AGFI = 0,801 5236 adolescents (grades 7–12) from 35 chinese schools					

	**Meinck al, 2020** [42] ICAST-C version; 42,194 schoolchildren; ages 11,13,16; from nine countries: Albania, Bosnia and Herzegovina, Bulgaria, Croatia, Greece, North Macedonia, Romania, Serbia, and Turkey. (n = 34,662; in Turkey, the sexual violence was not measured).			**Q: (−)** Kuder Richardson (KR20) command: good for exposure to physical violence (KR20 = .83) and psychological violence (KR20 = .84), adequate for neglect (KR20 = .69), poor for witnessing household violence (KR20 = .59) across all countries, and good for exposure to sexual violence (KR = .71) across eight countries (n = 34,662)		**Q: (−)** Standardized results of the total sample confirmatory factor analysis of the five-dimensional model among children: CFI = .964 TLI = 0.962 RMSEA = .021 SRMR = .075 (n = 34,662)			**Q: (** + **)** Configural model: CFI = .964 TLI = 0.962 RMSEA = .021 SRMR = .075. Metric model: CFI = .967 TLI = 0.966 RMSEA = .020 SRMR = .085. Scalar model: CFI = .956 TLI = 0.956 RMSEA = .023 SRMR = .083. Scalar 1 model: CFI = .956 TLI = 0.956 RMSEA = .023 SRMR = .083. (n = 34,662)		

**Sahaimi et al**, **2020** [43]			**Q: (−)** Alfa ranged from .59 to .77 in the five subscales. Overall alfa = ? 255 students 13–17 years of age were selected by a simple random sampling process.		**Q: (−)** Only the exploratory factor analysis was used: a total of 25 items were retained, and five factors were extracted.					

**E*vidence quality per measurement property***	**NA**		**QE: high**		**QE: high**			**QE: high**		

**DDI-C (form C)** *Dimensions of Discipline* *child self-report, form C* [[44], [45], [46], [47]] It provides current information on 26 of the most frequently used discipline behaviors of parents. It measures the frequency of using: • Corporal punishment; • Deprivation of privileges; • Diversion; • Explain/teach; • Ignore misbehavior; • Penalty tasks and restorative behavior; • Psychological aggression; • Reward; • Monitoring; • Context: conflict with partner, confidence, perceived ineffectiveness of disciplinary measures, stress • Sociodemographic risk scale.	**Straus and Fauchier, 2007[** [44,45]**]**	**Inadequate**^a^ The origin of the construct was clear, but the development study of form C (for children) was not performed in a sample representing the target population (pilot-testing with 53 parents and 499 university students reported the behaviors measured by the nine scales.									

	**Calvete et al, 2010 [46]** n = 1280 schoolchildren, ages 12–17^b^			**Q: (−)** Overall alfa = ? Alfa ranged from .39 to .81		**Q: (−)** RMSEA = .057 CFI = .97 NNFI = .97					

**Carvalho et al, 2017** [47] n = 190 schoolchildren, ages 12–17^b^			**Q: (−)** Overall alfa = ? Alfa ranged from .63 to 80		**Q: (−)** CFI = .91/.93 RMR = .250/.264 RMSEA = .10/.10 (for mothe**r/**for father in the four scales)					

***Evidence quality per measurement property***	**NA**		**QE:** **moderate**		**QE: moderate**					

**TISH** *Things I've seen and heard* [[48], [49], [50]] It measures current types of violence both witnessed and directly experienced by children, and examines the extent to which these exposure patterns are related to characteristics of the children and their families.	**Richters and Martinez**, **1993** [48] n = 165 children from Southeast Washington, DC, ages 6 to 10.	Original development tool work could not be accessed		**Not reported**	**Q** = **(** + **)** 1- week test-retest reliability with 21 children: r = .81 ICC or weighted Kappa not reported; sample <50.						

	**Hurt et al, 2001** [49]							**Q** = **(**±**)** Correlation with gold standard <0.70 and indirectness about target population in gold-standard instruments (Levonn instrument; CBCL-TRF)			

**Thompson et al, 2007** [50] 784 children from US (Eastern, Southern, Midwestern, Northwestern and Southwestern) completed a modified TISH scale at both ages 6 and 8.			**Q** = **(−)** Overall alfa = ? Alfa ranged from .58 to .74		**Q** = **(** + **)** CFA on age 8: CFI = .96 TLI = .97 RMSEA = .05 SRMR = .07 CFA on age 6: CFI = .96 TLI = .98 RMSEA = .05 SRMR = .05					

***Evidence quality per measurement property***	**NA**		**QE:** **moderate**	**QE** = **Low**	**QE: high**		**QE: moderate**			

**MNBS-CR** *Multidimensional* *Neglectful Behavior Scale- Child Report* [[51], [52], [53], [54]] It measures retrospective and current neglect of four basic developmental needs dimensions in four 5-item subscales: (1) neglect of physical needs such as food, clothing, shelter, medical care; (2) emotional needs such as affection, companionship, support; (3) supervisory needs such as setting limits, attending to misbehavior, knowing a child's whereabouts and friends; and (4) cognitive needs such as reading to the child, and explaining things.	**Straus et al, 2004** [51]	**Inadequate**^a^ Context and construct clearly described, but the preliminary version of the instrument was not performed in the target population.		**Q** = **(**±**)** Overall alpha: .93; short form A8 = .89; short form A4 = .81		**Q** = **(?)** CFA used, but not all information for good rating reported.					

	**Kantor et al, 2016** [52] 140 children (116 clinical, and 24 community, ages 10–15 and 177 children (114 clinical, 63 community), ages 6–9, from Maine and New Hampshire, US.	**Very good**^a^ Neglect data drawn from 200 child welfare–referred families. Authors held discussions with four child maltreatment forensic experts on areas of neglect, the proposed items, and strategies for interviewing children. A cognitive testing was conducted with 47 community children and 19 foster care children. Interviewer's written data about cognitive testing with children were reviewed by investigators.		**Q** = **(−)** Overall alfa: .69 (version for ages 6–9); .95 (version for ages 10–15).		**Q** = **(?)** Not clear which technique was used to reduce the number of items.					

	**Beyazıt and Ayhan, 2018** [53] 160 schoolchildren, ages 10–15, from Nicosia, Cyprus Island, Turkey.		**Adequate**^a^ Four professionals consulted the translated version Eight specialists for content validation Pre-pilot study (face validation): 13 children. Pilot study: 160 children. (not clear if all group meetings of interviews were recorded and transcribed, and the number of researchers involved in coding).	**Q** = **(** + **)** Mother and father forms analyzed independently: Overall alfa = .832 (mother form) Overall alfa = .908 (father form)		**Q** = **(** + **)** RMSEA = .048 (mother form) RMSEA = .041 (father form)		**Q** = **(−)** Correlation with gold standard (PARQ) < 0.70			

**Beyazıt and Ayhan, 2020** [54] 328 schoolchildren, ages 6–9, from Ankara and Antalya, Turkey.		**Adequate**^a^ Five specialists for content validation. Pre-pilot study (face validation): 10 children. Pilot study: 328 children. (not clear if all group meetings of interviews were recorded and transcribed, and the number of researchers involved in coding).	**Q** = **(** + **)** Mother and father forms analyzed independently: Overall alfa = .84 (mother form). Overall alfa = .85 (father form).		**Q** = **(** + **)** Mother form: RMSEA = .043 CFI >.95 Father form: RMSEA = .004 CFI = .99 (father form).					

***Evidence quality per measurement property***	**NA**	**QE** = **Moderate**	**QE** = **High**		**QE** = **High**					

**C-SARS** Checklist of *Sexual Abuse* *and Related Stressors* [55,56] It assesses victims' reports of the retrospective occurrence of 70 stressful events related to sexual abuse experiences. .	**Spaccarelli, 1995** [55] 48 sexually abused girls, ages 11–18, from Phoenix, Arizona, US	**Doubtful**^a^ The item development was guided by an alternative model that emphasizes abuse stress and coping in a transactional theoretical framework. The original development tool articles are unpublished and could not be accessed.		**Q** = **(−)** Overall alfa = .93 Evidence for sufficient structural validity unpublished.							

	**Wamser-Nanney et al, 2019** [56] 141 sexually abused children, ages 7–12, in a US Midwestern city			**Q** = **(−)** Overall alfa = .78							

***Evidence quality per measurement property***	**NA**		**QE** = **Low**							

**CEDV** *Child Exposure to Domestic Violence Scale* [[57], [58], [59], [60], [61]] It assesses current types of adult domestic violence to which a child is exposed, how a child is exposed to each, how the child is involved in violent incidents as well as information on other forms of victimization, and risk and protective factors.	**Edleson et al, 2007** [57] Pilot-testing with 46 children, ages 10 to 16.	**Adequate**^a^ The initial steps of tool development are described: a research team gathered question items from existing measures that use self-report by children up to 18 years old; the items were reviewed by an international panel of experts and piloted tested with 46 children aged 10 to 16. It is unclear about the use of the interview guide, transcription of the records, and approach used to analyze the data. .									
	**Edleson et al, 2008** [58] 65 children from domestic abuse community-based shelters or programs in the US, ages 10–16.	.	**Adequate**^a^ Interview guides based on key content areas identified in an earlier review; A panel of nine international expert judges working with children exposed to domestic violence was invited to review each item online and make suggestions. The final version was administered to 65 children from domestic abuse community-based shelters or programs. It is not clear about the methods to assess comprehensibility, data saturation and analysis.	**Q** = **(** + **)** Overall alfa = .84 Alfa ranged from = .50 to .76 in the six subscales.	**Q** = **(−)** 2-week test-retest period: Pearson's correlation coefficient for each subscale ranged from .57 to .70. ICC or weighted Kappa not reported.			**Q** = **(−)** Correlation with gold standard (TISH) < 0.70 Home violence exposure: r = .494, p < .001; community violence exposure: r = .397, p < .001.			

	**Harding et al, 2013** [59] 53 community children, ages 8–11, from the US.			**Q** = **(** + **)** Children's exposure to maternal and paternal perpetration of IPV was computed. Maternal IPV: alfa = .79; paternal IPV: alfa = .88).							

	**Grip et al, 2013** [60] 62 community children, ages 3–13, from Europe and outside Europe.			**Q** = **(** + **)** Children answered to two subscales on the tool; alfa were .79. and .73, respectively.							

**Ravi et al, 2019** [61] This systematic review included 13 studies to synthesize and summarize the psychometric properties of CEDV. Sample size ranged from 29 to 1212 children. The mean age of the children in the included studies was 12.70 (SD 3.30). Studies were conducted in US, Europe, Africa and Asia			**Q** = **(** + **)** Overall alfa >.7 in included studies. Subscales ranged from alfa 0.74 to 0.88.		**Q** = **(?)** None of the studies conducted a confirmatory factor analysis.		**Q** = **(?)** Significant positive correlation in concurrent validation with other instruments but not reported if > .70			

***Evidence quality per measurement property***	**QE** = **Moderate**	**QE** = **Moderate**	**QE** = **High**		**QE** = **Moderate**		**QE** = **Moderate**			

US = United States.

CFA: confirmatory factor analysis; CFI: comparative fit index; TLI: Tucker‐Lewis index.

RMSEA: root-mean-square error of approximation; SRMR: Standardized Root Mean Residuals; RMR: root-mean-squared residual.

GFI: goodness-of-fit index; AGFI: adjust goodness-of-fit index.

CTSPC: The Parent-Child Conflict Tactics Scale.

JVQ: Juvenile Victimization Questionnaire.

ICAST-C: International Association for the Prevention of Child Abuse and Neglect - Child Abuse Screening Tool.

DDI – C: Dimensions of Discipline child self-report, form C.

TISH: Things I've seen and heard.

MNBS-CR: Multidimensional Neglectful Behavior Scale- Child Report.

C-SARS: Checklist of Sexual Abuse and Related Stressors.

CEDV: Child Exposure to Domestic Violence Scale.

CBCL-TRF: Child Behavior Checklist, Total Report Form.

PARK: Parental Acceptance-Rejection Questionnaire.

MIMIC = Multiple Indicators, Multiple Causes (MIMIC) models.

**Q** = criteria for good measurement properties: **(** + **)** = sufficient (positive rating); **(?)** = indeterminate rating; **(−)** = insufficient (negative rating); **(**±**)** inconsistent rating.

**QE** = quality of evidence (GRADE): high; moderate; low; very low. Quality rating based on GRADE criteria: high level of confidence, moderate level of confidence, low level of confidence, very low level of confidence.

a Tool development is not a measurement property, it accounts when evaluating content validity. **NA**: Not applicable.

b Studies that analyzed psychometric properties in children aged 12 years and older were included if no articles on children under 12 years were found.

## 2 Results

**Fig. 3:**
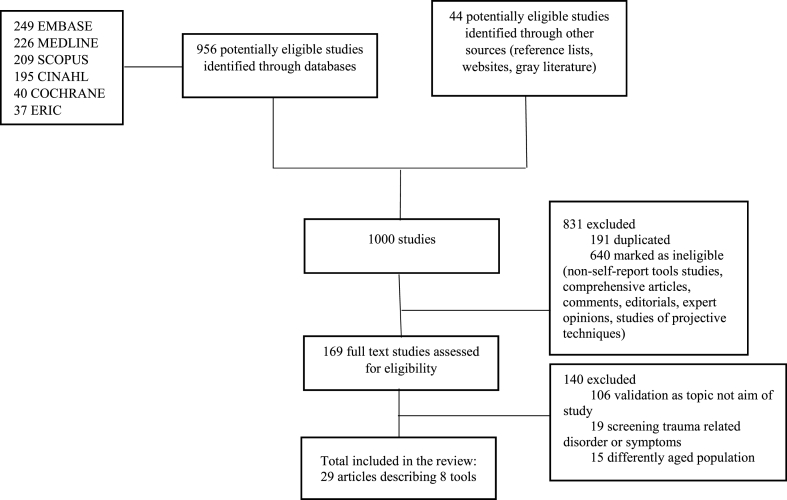
Flowchart of selection of studies.

This study identified, selected and assessed the eligibility of a total of 1000 articles, leading 29 studies representing eight child abuse measurement tools (Fig. 3.).

### 2.1 General characteristics of instruments

The tools selected were self-report instruments, self or interview administered. Specific studies developed to screen for the risk for CSA were not found, as authors did not encounter a risk conceptual framework or model. Studies addressing CSA exposure were then selected. Only studies in the English language were found. Most measures were developed in English, although some had been adapted and translated into other languages. The feasibility features of selected tools for children under twelve years of age are in Table 1. The eight tools used current, last year or lifetime perspectives for screening CSA.

We did not find an explicit emphasis on the psychogenetic aspects of child thinking development guiding the development of the instruments. The tools developed from the 2000s onward regarded more the aspects about comprehensibility and comprehensiveness, where it was easier to identify quality parameters for content validity. New studies conducted in recent years with tools developed in the 1990s have allowed for more transparent evaluation of quality criteria that were not possible in the original studies. None of the eight tools identified could be assessed for all nine COSMIN criteria because no information related to measurement error or responsiveness were found. The strength of evidence for each tool, per measurement property was summarized in Table 2.

Certain properties were reported as undetermined (?). This is justified for two reasons: not finding the described property in the consulted papers; not accessing the referenced papers or clarifications, even after attempts to contact, by email, authors or regulatory agencies of the tools to provide additional information.

### 2.2 Comparative assessment of instruments

Table 1, Table 2 shows comparative aspects between instruments. ICAST-C was the tool that obtained the highest number of rated measurement properties and strength of evidence, followed by: JVQ, MDNB-CR, DDI, TISH, CEDV, C-SARS and CTSPC. CTSPC, C-SARS and CEDV had no structural validity studies.

The ICAST-C was developed by a team of international experts on child abuse research for children aged 12–17; in some pilot tests a small number of children were less than 12 y and there are child versions for ages 11–18 y. It was tested in China in 12–14 years old adolescents and indicated good internal consistency of the 50-item ICAST–CH–C and *α*′s for the five subscales ranged from 0.71 to 0.89. Multigroup confirmatory factor analyses of 42,187 children in nine Balkan countries was satisfactory.

The JVQ [[36], [37], [38], [39]] was developed in interview format for children from 8 to 17 years old, with 40 items, with an adequate study design, but we did not find any report on content validity testing. MDNB-CR [[51], [52], [53], [54]] has gained in methodological quality and in the number of studied measurement properties studied since 2016.

A sociodemographic risk scale exists in DDI form-C. DDI-C could be considered a domestic CSA risk instrument for regarding other risk factors as parent-child relationship and psychological/physical violences [[44], [45], [46], [47]]. The TISH [[48], [49], [50]] showed good feasibility, however it was not possible to evaluate the TISH on the quality of tool design or content validity because of the absence of such information in the published papers and because contact with the authors was not possible.

A specific theoretical model for the development of the CEDV construct and items was not described in the first published paper [58], and it was noted that the authors collected items from existing content-based interview questions and guides identified in a previous review [57]. In the cited review, the tools identified were CTSPC, TISH, JVQ, the Victimization Scale (Nadel et al., 2006 *apud* Edleson et al., 2007), developed for adolescents from the age of 12 (sixth to eighth grade), with 135 questions on domestic (12 items) and community/school victimization (123 items), and the Violence Exposure Scale for Children - Revised (Fox and Leavitt, 1996 *apud* Edleson et al., 2007) restricted to book publication, where access was not possible for us through the bibliographic bases, as well as no published works were identified.

C-SARS [55,56] presented the lowest number of eligible studies among the eight instruments and only one measurement property studied (internal consistency). The first tool for use in child-report was CTSPC [[33], [34], [35]], which had wording adapted for children aged 6–17 years, but showed insufficiency in the general criteria for tool design and content validity. The adult versions of the tool have been more extensively studied and have better detailed measurement properties than the children's versions. ^34^A Sinhala versions' internal consistencies were superior to those of its original version, but it was validated only on 12-year-old Sinhala schoolchildren [35].

## 3 Discussion

This research aims to contribute to the scientific field, mainly by identifying the strengths and weaknesses of under-12 y children self-report instruments for CSA risk or exposure detection in the domestic context. It focuses on screening CSA pre- and post-exposure settings still in childhood. We did not include tools focusing on CSA trauma or sequelae. This preventive approach could be a strategy to follow the United Nations 2030 Agenda for Sustainable Development [64].

Eight self-report tools were assessed. We observed that theoretical conceptions structured in models or descriptor matrices were rare, but instrument development was adequate or very good for five tools. We would have liked to have found an expressive emphasis on the psychogenetic developmental aspect of children's thinking, which does not seem to have been the focus of the development of the instruments. The instruments showed methodological heterogeneity. No single instrument reported all nine measurement properties outlined by the COSMIN methodology. Measurement error, responsiveness, and hypothesis testing were the most rarely reported. Three tools still need structural validity studies.

This review has several strengths. The pre-registered protocol ensured that inclusion and exclusion criteria were determined in advance. Two or three reviewers conducted the assessment for screening, extraction, and quality. Manuscript adjustments recommended by peer review were concurrent with the participation of this review in the PRISMA-COSMIN guideline pilot testing [63].

Limitations of this review include restrictions on obtaining additional information from developers, books, and unpublished articles. In addition, it seemed challenging to apply 2018 COSMIN criteria to instruments that have been developed since the 90s. Nevertheless, most studies showed compliance with many of the quality parameters.

None of the tools used a specific risk measurement construct for CSA in this age group. It is possible to infer that risk is, per se, a complex theoretical construct. Its theoretical complexity is in evolution along the path of science itself. There are significant qualitative advances in its methodological dimension but with a need to integrate its theoretical complexity into the paradigm of health promotion currently in place. Promoting health needs an integrated and complex theoretical-methodological framework in scientific and operational development, and it is still unequal in several countries.

The domestic context would be the most implicated as the emerging starting point of the various forms of violence. Identifying the sexual component of domestic violence would increase the chance of detecting other forms that would constitute risk factors for CSA, such as neglect, physical, psychological, and community violence. The predictive analysis of contexts and behaviors in the domestic environment is one of the tasks that transcend disciplinary boundaries and is not only the responsibility of Pediatrics, Psychology, Epidemiology, Education, and Social Work but of a complex network of scientific and operational, interdisciplinary, and intra/intersectional cooperation.

The literature provides evidence of co-occurrence of forms of violence. Intergenerational is one of the most significant risk factors for CSA. However, it was not observed to be addressed in items of the reviewed tools. Mohr and Tulman’ (2000) and Edleson et al. (2007) reinforced the importance of self-report multidimensional assessment tools for domestic violence that should include a level of risk and protective factors, co-occurrence of child maltreatment, and the child's coping abilities. Those tools bring benefits in terms of epidemiological assessment, application of monitoring measures, and rehabilitation for children and their families. The secondary prevention of risks and the early detection of harm to the biopsychosocial and ecological health of children and their families is imperative.

Children's participation as co-constructors during the development of the instruments is relevant. Tay-Lim and Lim (2013) argued that from early childhood, children could have their voices empowered in the co-construction of knowledge and worldview with children's eyes from the employment of participatory methodologies in research. Hence, children would be able to share their opinions about issues that concern them, making sense of their views. They would be active, competent, and reflective builders of their own world experiences and would be able to act as social and political protagonists on what affects them directly [62].

Tools that are more adapted to cognition by age group, that consider time of completion, and that have been developed with the active participation of children in face and content validation seem more appropriate in the aspect of feasibility. Stratifying the theoretical models by age, considering the psychogenetics of children thought proper to the age groups, could also increase the tool's power of accuracy. It was observed that the tools developed after 2000 presented playful elements, such as figures and answer options that included the possibility of painting. The recent use of drawings, emojis, and technology for computerized versions of the tools may be features that will facilitate child compliance.

## 4 Conclusion

The present article is the first COSMIN systematic review to assess the psychometric properties of self-report instruments for children under 12 to detect risk or exposure to CSA in a domestic context. Our preventative approach does not include instruments focused on CSA trauma or sequelae. This review assesses a total of 29 studies representing eight measurement tools. No single instrument reported all nine measurement properties outlined by the COSMIN methodology. The strength of evidence is moderate to high for six instruments with the highest number of rated psychometric properties. None of the tools were found using a specific risk measurement construct for CSA for under-12 children. We suggest that future measurement instrument developers consider further studies of the complexity of the CSA risk construct by age group to psychometrically assess and prevent the intergenerational sexual violence cycle.

## Data availability statement

Data included in: https://drive.google.com/drive/folders/1MrwBhlXNEQGals2aUWcpREJPXdr039yi?usp=sharing.

## CRediT authorship contribution statement

**Karla Danielle Xavier do Bomfim:** Conceptualization, Data curation, Formal analysis, Investigation, Methodology, Project administration, Resources, Software, Validation, Visualization, Writing – original draft, Writing – review & editing. **Umbelina do Rego Leite:** Conceptualization, Data curation, Formal analysis, Investigation, Methodology, Project administration, Resources, Supervision, Validation, Visualization, Writing – review & editing. **Paulo Sávio Angeiras de Goes:** Conceptualization, Data curation, Formal analysis, Investigation, Methodology, Project administration, Resources, Software, Supervision, Validation, Visualization, Writing – review & editing.

## Declaration of competing interest

The authors declare that they have no known competing financial interests or personal relationships that could have appeared to influence the work reported in this paper.
